# Optimization of HS-SPME for GC-MS Analysis and Its Application in Characterization of Volatile Compounds in Sweet Potato

**DOI:** 10.3390/molecules26195808

**Published:** 2021-09-25

**Authors:** Rong Zhang, Chaochen Tang, Bingzhi Jiang, Xueying Mo, Zhangying Wang

**Affiliations:** Crops Research Institute, Guangdong Academy of Agricultural Sciences & Key Laboratory of Crop Genetic Improvement of Guangdong Province, Guangzhou 510640, China; zr9362@126.com (R.Z.); tangchaochen1988@163.com (C.T.); jiangbz2004@163.com (B.J.); mxy68269@163.com (X.M.)

**Keywords:** sweet potato, volatile compounds, HS-SPME, GC-MS

## Abstract

Volatile compounds are the main chemical species determining the characteristic aroma of food. A procedure based on headspace solid-phase microextraction (HP-SPME) coupled to gas chromatography-mass spectrometry (GC-MS) was developed to investigate the volatile compounds of sweet potato. The experimental conditions (fiber coating, incubation temperature and time, extraction time) were optimized for the extraction of volatile compounds from sweet potato. The samples incubated at 80 °C for 30 min and extracted at 80 °C by the fiber with a divinylbenzene/carboxen/polydimethylsiloxane (DVB/CAR/PDMS) coating for 30 min gave the most effective extraction of the analytes. The optimized method was applied to study the volatile profile of four sweet potato cultivars (Anna, Jieshu95-16, Ayamursaki, and Shuangzai) with different aroma. In total, 68 compounds were identified and the dominants were aldehydes, followed by alcohols, ketones, and terpenes. Significant differences were observed among the volatile profile of four cultivars. Furthermore, each cultivar was characterized by different compounds with typical flavor. The results substantiated that the optimized HS-SPME GC-MS method could provide an efficient and convenient approach to study the flavor characteristics of sweet potato. This is the basis for studying the key aroma-active compounds and selecting odor-rich accessions, which will help in the targeted improvement of sweet potato flavor in breeding.

## 1. Introduction

Sweet potato (*Ipomoea batatas* L.) is one of the most important crops in the world and ranks fifth in caloric contribution to the human diet [1]. China is the largest producer of sweet potato in the world, with an annual production of 52 million tons that accounted for 57% of the world’s production in 2019 [2]. Sweet potato has been gradually transformed from a main food into health and special food since it is rich in many nutritional and functional components [3]. In China, sweet potato has a wide range of applications such as table use, processed food, starch production, and animal feed. In recent years, the proportion for table use consumption has gradually increased (about 40–50% of total consumption), and the released cultivars for table use accounts for 56.6% of all bred varieties [4,5]. In addition to yield, resistance, and good appearance, the most important breeding objective for table use is good eating quality. Flavor directly affects the eating quality and the choice of consumers, and therefore acts as an important quality index to be considered in the breeding programs of sweet potato.

Generally, food flavor consists of aroma, taste, and sense, among which aroma is essential for good flavor [6]. Humans are sensitive to aroma and can perceive up to 10,000 distinct odors. Food aroma is determined by the combination of volatile compounds [7]. However, the evaluation of volatile compounds is time-consuming, costly, and impressionable, which has led to most breeders focusing on yield, disease and pest resistance, and starch content rather than aroma trait. The volatile compounds have largely been ignored, which leads to the lack of high-quality sweet potato cultivars with different aroma in the market. Identifying the volatile composition of sweet potato is the starting point to reveal the formation mechanism of aroma compounds and to further improve the flavor quality of sweet potato.

The volatile compounds can be separated and analyzed as individual compounds using GC depending on the volatiles extracted from the matrix. Thus, the accurate extraction of volatiles is critical for the whole determination [8]. Different extraction methods have been used to analyze volatiles in food including sweet potato such as the purge and trap technique [9,10], steam distillation (SD) [11,12], simultaneous distillation extraction (SDE) [13], and solid phase microextraction (SPME) [14,15]. The purge and trap technique and SD were used for identification, but not suitable for quantification. SD and SDE based on distillation require a large quantity of samples, a long-time, and organic solvents. Furthermore, these methods frequently separate non-volatile chemical compounds interfering with the analysis and increase the detection limits, causing the huge difference in the results obtained in different studies [16]. Headspace solid phase microextraction (HS-SPME) is a rapid, simple, and solventless method for volatile extraction since it combines sampling and sample extraction in one step [17]. HS-SPME combined with GC-MS has been demonstrated to be a useful tool in identifying volatile compounds in a wide variety of foods like fruits [18], teas [19], crops [20], and meat products [21]. It is also used to compare the relative amounts of the volatile compounds among samples when the same analytical procedure is used [22,23]. Recently, it has been successfully used for determining the volatile flavor profiles of baked sweet potato. More than 100 volatile compounds were identified and aldehydes, alcohols, ketones, and terpenes were the main components. Compounds such as phenylacetaldehyde, benzaldehyde, maltol, β-ionone, and more than another 10 compounds were identified as the main odor-active compounds of baked sweet potato [14,15,24]. However, in these previous studies, the samplings were cooked sweet potato and then reheated during incubation to promote the extraction of volatile compounds. As is well-known, during the cooling of the cooked sweet potato, its aroma almost disappears and is not as strong as before when reheated. Therefore, the attractive flavor produced by the thermal reaction was difficult to reproduce and capture.

In this study, we aimed to develop an analytical method combining HS-SPME and GC–MS to detect the volatile components closer to the real flavor of sweet potato. To achieve this aim, experimental conditions were optimized by testing different types of SPME fibers, incubation temperature and time, and headspace time in order to utilize sufficient incubation temperature and time to reach the cooked and equilibrium state and avoid the loss of volatiles with reheated samples. Then, the optimized method was used for the identification and quantification of volatile compounds of four sweet potato cultivars. The methodology based on HS-SPME could provide a good starting point to investigate the key aroma activity compounds of sweet potato and lay the foundations for flavor improvement in the sweet potato breeding program.

## 2. Results and Discussion

### 2.1. Fiber Selection

**Figure 1:**
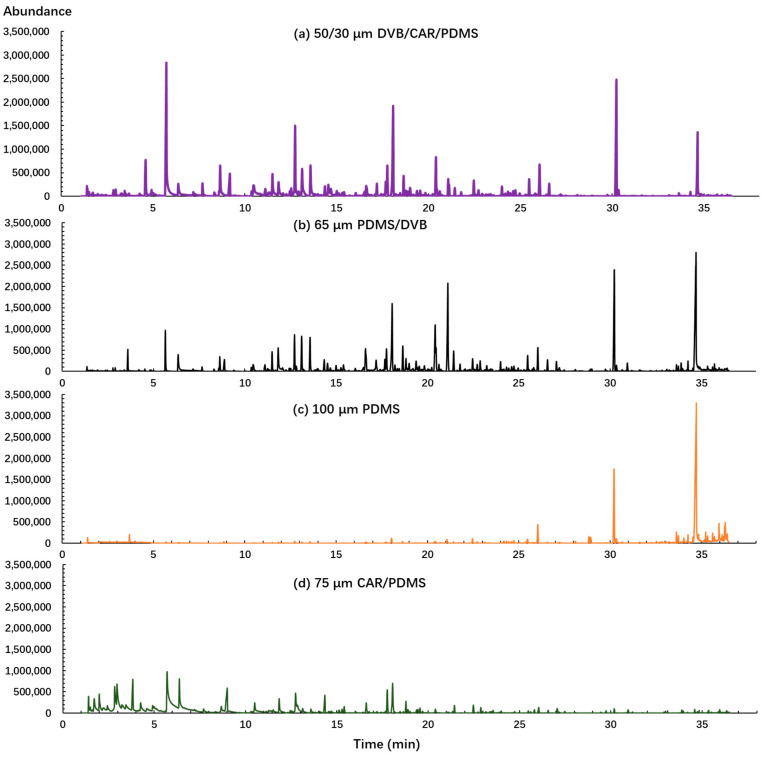
Chromatograms obtained from four different fibers (DVB/CAR/PDMS, divinylbenzene/carboxen/polydimethylsiloxane; PDMS, polydimethylsiloxane; PDMS/DVB, polydimethylsiloxane/divinylbenzene; CAR/PDMS, carboxen/polydimethylsiloxane) using the same sample and with the same optimized condition.

First, the fiber coating was evaluated in this study because it is one of the most important factors in determining the quality and quantity of detected volatile compounds. Four different types of SPME fibers (PDMS, CAR/PDMS, PDMS/DVB, DVB/CAR/PDMS) were tested using the same sample and analysis conditions. The largest number of identifiable peaks and mass spectrometry (MS) peak areas were used to evaluate the performance of SPME fibers. As shown in Figure 1, it is evident that different fiber coatings have significant effects on the extraction of volatile compounds from sweet potato. Both DVB/CAR/PDMS and PDMS/DVB enabled the detection of a wider range of compounds and produced a higher peak intensity than the other two fibers. PDMS provided the highest responses of large molecular weight compounds captured after 30 min and CAR/PDMS absorbed the largest number of low molecular weight compounds captured before 10 min. In total, 56, 49, 44, and 29 volatile compounds, and 8.75 × 10^7^, 8.26 × 10^7^, 3.66 × 10^7^, and 6.23 × 10^7^ total chromatographic areas were identified by DVB/CAR/PDMS, PDMS/DVB, PDMS, and CAR/PDMS fibers, respectively. Since the PDMS and CAR/PDMS fibers obtained less volatile compounds and MS peak area, they were not considered for further steps. Though PDMS/DVB fibers captured a similar number of volatiles and total chromatographic area as those of the DVB/CAR/PDMS fiber, more undesired high intensity impurity peaks and lower match factors were detected using PDMS/DVB than the DVB/CAR/PDMS fibers. Thus, the DVB/CAR/PDMS fiber was selected as the best fiber coating for further analysis.

These results are consistent with the characteristics of fibers. PDMS fiber is a nonpolar fiber designed for volatiles and semi-volatiles with large molecules and CAR/PDMS is a bipolar fiber, which is more suitable for gases and low molecular weight compounds [25]. The PDMS/DVB fiber is inclined to extract semi-volatile compounds and large volatile molecules and it has been reported as the best option for many polar analytes such as pesticide residues [26]. The fiber with the DVB/CAR/PDMS coating combines the characteristics of three different absorbing phases (PDMS, DVB, and CAR), and can trap a wide range of volatile components with different volatility, polarity, structure, and molecular weight. The DVB/CAR/PDMS fiber was identified as the optimal fiber and provided good results in qualitative and quantitative analysis for many food and products such as oranges [27], rice [28], red and white wine [29], and potato products [30,31]. Consequently, the fiber with a DVB/CAR/PDMS coating was selected as the best fiber coating for the identification of sweet potato volatile compounds.

### 2.2. Incubation Temperature and Time

**Figure 2:**
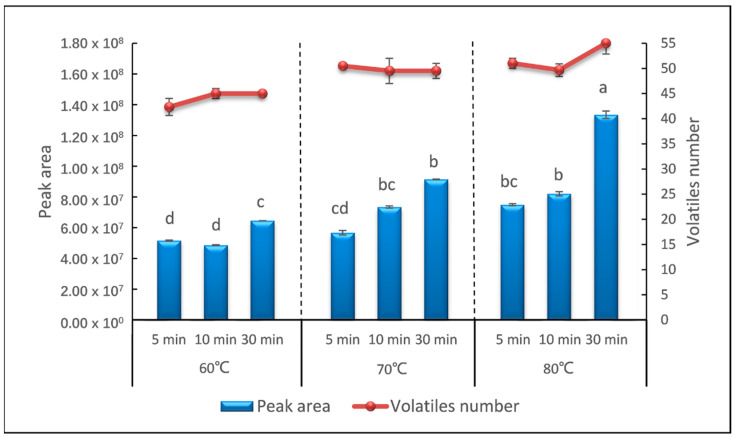
Peak area and number of volatiles detected in different temperature and time of incubation. Means with different lowercase letters indicate significant difference with *p* < 0.05.

The incubation temperature and time affect the extraction efficiency and selectivity of sweet potato volatiles into the headspace. In this study, the sweet potato samples were heated in the process of incubation to reach the cooked and equilibrium state. A factorial design was performed to evaluate the effect of incubation temperature and incubation time to the sweet potato samples. Three incubation temperatures of 60 °C, 70 °C, and 80 °C, and three incubation times of 5 min, 10 min, and 30 min were investigated. It could be easily seen that with the increase in the incubation temperature and incubation time, both the total peak area and the number of identified volatile compounds gradually increased (Figure 2). The total peak area increased from 5.1 × 10^7^ (60 °C, 5 min) to 1.36 × 10^8^ (80 °C, 30 min) and the number of identified compounds increased from 42 (60 °C, 5 min) to 55 (80 °C, 30 min). Then, the identified compounds were analyzed. The majority of analytes (95%) detected in 5 min at 60 °C gave a relative standard deviation (RSD) below 20%. When the temperature increased to 70 °C and incubated for 30 min, RSDs of the relative content of 52.7% analytes were lower than 20%. The volatile compounds released by the samples incubated at 80 °C for 30 min were more stable, and 88.7% analytes gave a RSD lower than 20% (data not shown).

The extraction efficiency increased with temperature and time, and was the highest at 80 °C for 30 min, the upper bound of our incubation parameter. Furthermore, strong sweet and honey notes were released by the samples incubated at this condition, which is similar to the smell of cooked sweet potato. To verify whether 80 °C for 30 min was the most suitable incubation condition, we extended the incubation time to 50 min and 60 min at an incubation temperature of 80 °C. As a consequence, the total peak area captured was 1.30 × 10^8^ and 1.02 × 10^8^, and the number of identified compounds were 54 and 53, respectively (data not shown). The extracted compounds and extraction precision were mostly the same as that incubated for 30 min. Incubation temperatures higher than 80 °C were not tested further since this temperature is enough for the starch of sweet potato to gelatinize. The aldehydes could already been observed as the most abundant volatile compounds in sweet potato samples and the higher temperature could have a strong modification on the pattern of aldehydes [8]. On the other hand, the progressing of analytes absorbed from headspace onto a fiber coating is an exothermic process, which can be hindered by high temperature and the partition of analytes would also be impacted [32]. Hence, incubation at 80 °C for 30 min was selected for further analysis.

### 2.3. Extraction Time

Extraction is the progressing of analytes achieving distribution equilibrium between the sample matrix, headspace, and the coating of the fiber, and a sufficient extraction time was used to achieve the equilibrium [33]. The coverage and content of captured volatile compounds could be affected by the extraction time [34]. Therefore, extraction time of 30, 40, and 50 min were studied for samples incubated at 80 °C for 30 min; the extraction temperature persisted at the incubation temperature. The result showed that 1.36 × 10^8^, 1.60 × 10^8^, and 1.71 × 10^8^ total chromatographic peak areas were obtained by extraction times of 30 min, 40 min, and 50 min, respectively. The number of identified volatile compounds were similar (58, 56, 56), but more compounds with higher molecular weight (>200) were detected under the extraction times of 40 min and 50 min (nine for 30 min, 19 for 40 and 50 min) and the chromatographic area proportion of these compounds had a substantial increase from 17.63% (30 min) to 25.64% (40 min) and 35.68% (50 min). Most were products of the thermal oxidation of fatty acids and fatty acid derivatives, which are odorless compounds or have slight odor with very high odor threshold value, with few effects on the aroma flavor of samples, but the high abundance of them probably prevents the adsorption of target analytes [35]. Consequently, the 30 min extraction condition was employed since it provided a superior balance between extraction efficiency and selectivity of analytes.

Overall, the optimum HS-SPME GC-MS experimental conditions for sweet potato volatile compounds analysis were fixed as: the DVB/CAR/PDMS coating fiber, incubation at 80 °C for 30 min, and extraction at 80 °C for 30 min.

### 2.4. Profiling of Volatile Compounds in Sweet Potato

#### 2.4.1. Identification of Volatiles in Sweet Potato

Four sweet potato cultivars with different flavor (Table A1 in Appendix A) were selected and subjected to GC–MS analysis under the optimized experimental parameters. The volatile compounds identified in the four sweet potato cultivars are shown in Table 1. The ANOVA analysis illustrated the significant differences among the volatile profiles of the different cultivars. In total, 68 volatile compounds were identified in these four cultivars by the mass spectra matched to NIST2017 with a match quality score ≥ 80, among which 28 compounds have been reported in baked sweet potato [10,11,36,37,38] and the other 40 volatile compounds were identified for the first time in sweet potato. Around 80% compounds in each cultivar produced a RSD value below 20%, indicating that the optimized method has satisfactory repeatability. According to their chemical structure, identified compounds were classified as aldehydes (21), alcohols (13), terpenes (eight), ketones (11), acids (three), phenols (two), alkanes (two), esters (two), furan (one), ethers (one), and others (four).

**Figure 3:**
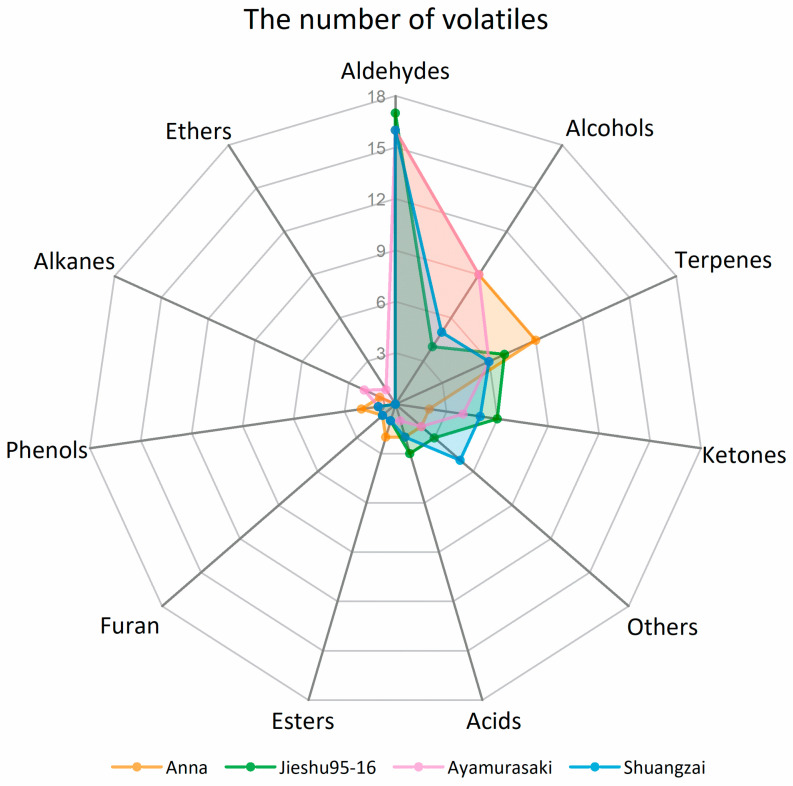
Classes of volatile compounds in the four sweet potato cultivars (Anna, Jieshu95-16, Ayamurasaki, and Shuangzai).

The number and classes of identified volatile compounds and their relative concentrations in the different cultivars varied. In total, 48, 45, 47, and 42 volatile compounds were identified in Anna, Jieshu95-16, Ayamurasaki, and Shuangzai, respectively. The number of volatile compounds in each chemical class in the four cultivars are demonstrated in Figure 3. Aldehydes were the most numerous volatile compounds followed by alcohols, ketones, terpenes, and acids, contributing to around 90% of the volatile compounds of sweet potato. The numbers of aldehydes in the four cultivars were similar while Anna and Ayamurasaki had enriched alcohols. Furthermore, a significantly higher number of terpenes were identified in Anna. Jieshu95-16 and Shuangzai presented similar compositions of volatile compounds classes, and both showed higher levels of ketones and acids. The relative quantitative results showed that Anna contained significantly higher concentration of alcohols, terpenes, and acids while Jieshu95-16 and Shuangzai showed a significant higher content of aldehydes and Shuangzai showed a higher content of ketones. The concentration of different class of compounds in Ayamurasaki was moderate (Table 2).

#### 2.4.2. Aldehydes

Thirteen aldehydes were identified as the common compounds in the four cultivars, among which hexanal and (E)-2-heptenal were described as grass and soap flavor, respectively, showing similar levels in the four cultivars. All the other aldehyde compounds were significantly different. The content of benzeneacetaldehyde, characterized by an attractively sweet and honey odor, was abundant in Ayamurasaki. Benzeneacetaldehyde is the most abundant compound detected in sweet potato, which was one of the most potent odorants in baked ‘Jewel’ sweet potatoes with the highest flavor dilution (FD) value [38]. This is produced from the aromatic amino acid phenylalanine (Phe) degradation [40,41]. From the same origination, benzaldehyde, with its odor linked to pleasant sweet and caramel odor, also showed a higher level in Ayamurasaki than in the other cultivars. 2,4-Dimethylbenzaldehyde, a cherry and vanilla flower flavor contributor [42], was produced from benzaldehyde and was only detected in Anna. Thus, we may conclude that in this study, aldehydes derived from the Phe contributed to the fragrant and sweet flavor of sweet potato, especially in Ayamurasaki.

(E,E)-2,4-Decadienal and (E)-2-octenal were also predominant components among the aldehydes, which were considered as aroma contributors to the baked sweet potato [9,10,24]. (E,E)-2,4-Decadienal is the second most abundant compound in Jieshu95-16 and Shuangzai, significantly higher than that in Ayamurasaki. A high amount of (E,E)-2,4-decadienal gave an unpleasant flavor with fat, greasy, and rancid notes since it presented a very low odor threshold (0.07 ppb) [43]. Thus, the unpleasant heavy oily flavor produced by (E,E)-2,4-decadienal might have a greater effect on Jieshu95-16 and Shuangzai than Ayamurasaki. (E)-2-Octenal, which contributed an unpleasant green and fatty flavor note to samples, was more abundant in Shuangzai. Both (E,E)-2,4-decadienal and (E)-2-octenal mainly originated from the autoxidation of oleic acid, which is involved in the process of fatty acid oxygenation [44]. Fatty acid degradation products associated with green leaf or fat flavor like (E)-2-hexenal, nonanal, (E,E)-2,4-nonadienal [45], and some other derivatives provided fruity or fat odor notes such as (E)-2-pentenal, heptanal, (E,Z)-2,4-decadienal, and (E,E)-2,4-heptadienal. Among these compounds, nonanal (fat, citrus, green) and (E,E)-2,4-nonadienal (fat, wax) have been reported to be active contributors of flavor in baked sweet potato and other compounds were not reported in sweet potato. In the present study, the relative contents of 2-hexenal (green), (E,Z)-2,4-decadienal (fried, wax), and (E,E)-2,4-heptadienal (nut, fat) in Jieshu95-16 were significant higher than that in Anna and Ayamurasaki. Thus, most aldehydes produced by fatty acid oxidation in this study were generally related with unpleasant green, fatty and wax odor. The content of these compounds in Ayamurasaki and Anna were generally lower than that in Jieshu95-16 and Shuangzai. It was suspected that fatty acid oxidation products contributed a more unpleasant flavor for Jieshu95-16 and Shuangzai than for Ayamurasaki and Anna.

#### 2.4.3. Alcohols

Alcohols were the second largest classes of identified volatile compounds in sweet potato, especially in Anna and Ayamurasaki. As indicated in Table 2, amongst all the volatile compounds in four sweet potato samples, 13 alcohols were identified. Three compounds—1-hexanol, terpinen-4-ol, and (−)-myrtenol—were identified as common compounds in all cultivars. Terpinen-4-ol has been reported as common aroma compounds in baked sweet potato [14]. The terpinen-4-ol odor is described as a typical turpentine, nutmeg and musty. Shuangzai stood out as having a significantly higher value for terpinen-4-ol, which was suspected to have an adverse effect on aroma.

The most abundant alcohol in Anna and Ayamurasaki was *p*-cymen-7-ol, which related to a caraway-like and herb flavor. Benzyl alcohol and phenylethyl alcohol, which were also derived from the Phe, showed higher levels in Anna than in the other cultivars and provided a delightful aroma like a rose flavor and honey sweet flavor [42]. Perilly alcohol appeared in Anna and Ayamurasaki, and has been reported in the essential oil of boiled Ayamurasaki [38] where the limonene degradation produces the orange and floral flavor. It is easy to discover that most of the identified alcohols presented flowery, fresh, and sweet flavor notes and accumulated in Anna and Ayamurasaki more than in Jieshu95-16 and Shuangzai. Hence, it is suggested that the alcohols identified in this study contributed a pleasant aroma for sweet potato and had a greater effect on the aroma of Anna and Ayamurasaki.

#### 2.4.4. Ketones

Ketones have a relative high variety specificity in sweet potato, and no common ketones were detected in all cultivars. Trans-β-ionone and 6-methyl-5-hepten-2-one are the reported ketones in baked sweet potato [14,38]. Trans-β-ionone, the degradation product of carotenoids, was detected in Shuangzai and Anna. 6-Methyl-5-hepten-2-one, produced by the degradation of lycopene, was uniquely identified in Shuangzai. This can probably be explained by the fact that Shuangzai has an orange flesh color, which had a high level of carotenoid [46], and the light yellow flesh colored cultivar Anna contained different types of carotenoid than Shuangzai.

#### 2.4.5. Terpenes

Terpenes are another important aroma contributor for plants since it constitutes the largest and most diverse class of secondary metabolites. Terpenes, containing monoterpenes, diterpenes, and sesquiterpenes, are derived from isopentenyl diphosphate (IPP) and dimethylallyl diphosphate (DMAPP) [47,48,49]. In the present study, eight terpenes were identified. Camphene with a typical camphoraceous flavor was abundant in all cultivars and particularly in Anna and Ayamurasaki, which contained four or five times that of the other cultivars. Nerol was identified as a common compound in all cultivars and has been reported as common aroma compounds in baked sweet potato. Anna had the highest level of nerol, which provided a pleasant sweet and lemon flavor, followed by Shuangzai, while Jieshu95-16 reported the lowest content. Geraniol, one of the most potent odorants in baked sweet potato [24], had a high level in Ayamurasaki, but was not identified in Shuangzai. Its odor was characterized as rose and geranium. Two woody flavor compounds, α-huaiene and humulene, were characteristic sesquiterpenes of Anna; both were first reported in sweet potato. β-Selinene with a herbal flavor appeared only in Jieshu95-16. Most of the terpenes are present in trace amounts, however, in some cases, low-abundance terpenes stand out as important flavor active compounds since most terpenes have low odor threshold values [50].

#### 2.4.6. Other Compounds

In the four examined sweet potato cultivars, one furan, two phenols, and three acids were identified. Furans are believed to result from Maillard reactions. 2-Pentyl-furan with a green and fruity odor was the unique furan and was detected in all samples. Guaiacol, giving a smoky, sweet and coffee odorant, was found to be abundant in all four cultivars, especially in Ayamurasaki. It was the strongest aroma compound for boiled sweet potato oil [38] and is also well-known in many processed foods such as cooked black rice, tea, roasted coffee, and wine [51,52,53,54]. Large amounts of *n*-hexadecanoic acid were detected in all samples while it had few contributions to the aroma flavor. Trace numbers and amounts of alkanes, esters, and other compounds were identified in sweet potato. Almost all of them were first reported in sweet potato, and may be formed during the thermal degradation of glucose or the degradation of fatty acids by lipoxygenase.

### 2.5. Multivariate PCA and PLS-DA Analyses

**Figure 4:**
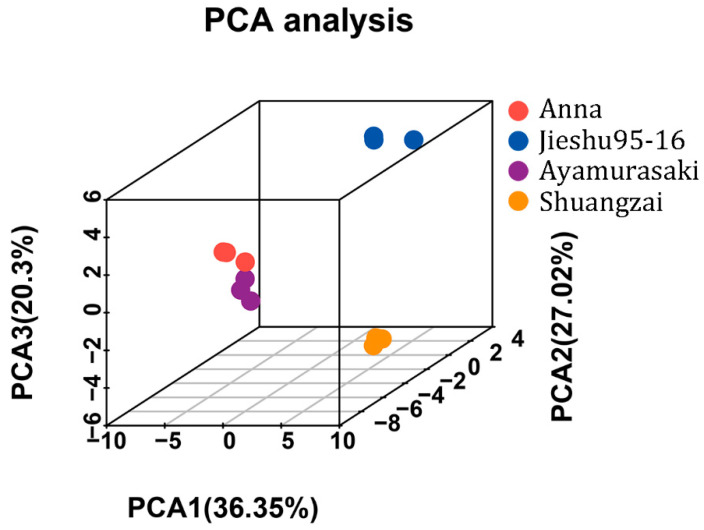
Principal components analysis results of the four different cultivars of sweet potato.

The unsupervised principal components analysis (PCA) score plot of all volatile compounds (Figure 4) showed that replicates of the individual cultivar showed good repeatability, which again confirmed the feasibility of the optimized HS-SPME GC-MS method. Furthermore, according to the results of PCA, the samples of four cultivars were distributed into separate groups based on the first three principal components, indicating that the cultivars had significant impact on volatiles. The PCA score plot showed 36.35%, 27.02%, and 20.3% variances by PC1, PC2, and PC3, respectively. Anna and Ayamurasaki were closely located, indicating that they probably have a similar flavor, which is consistent with the sensory analysis results as both of them were evaluated as having higher overall taste and presented sweetness and a caramel taste. In contrast, Jieshu95-16 and Shuangzai were far from each other, which means they have significantly different flavors.

**Figure 5:**
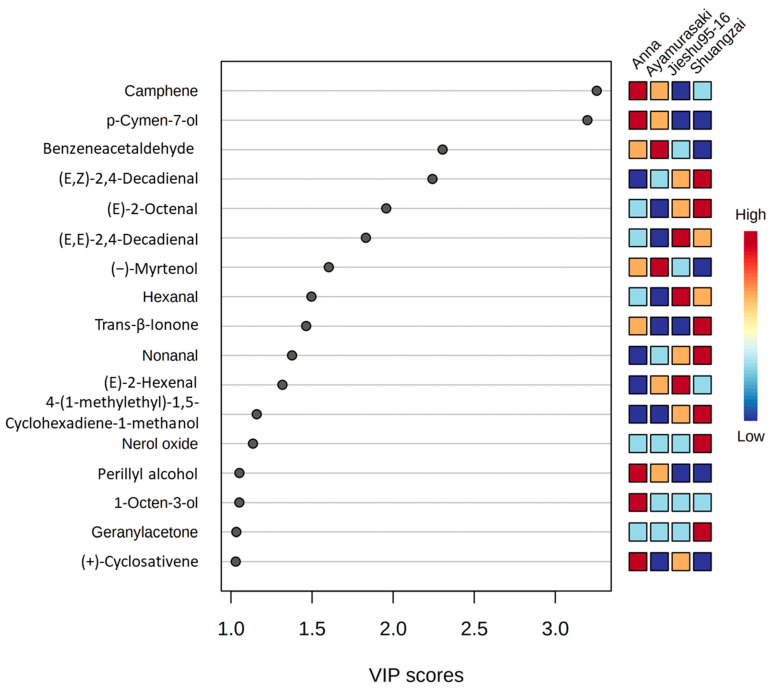
Partial least squares-discriminate analysis (PLS-DA) score plot of the GC-MS data. A variables important in projection (VIP) score close to or greater than 1 of the compound was considered to be a differential volatile in the cultivars.

The PLS-DA model was applied to distinguish between the four cultivars for the GC-MS results. The variable importance projection (VIP) method was used to explore the characteristic volatile metabolites in the PLS-DA model. In the VIP score plot (Figure 5), 17 compounds appeared to exhibit a VIP value greater than 1, indicating that these volatile compounds might play as differential volatiles for four cultivars in the PLS-DA model. Among them, camphene, *p*-cymen-7-ol, perillyl alcohol, 1-octen-3-ol, and (+)-cyclosativene could be considered as differential volatiles for Anna. The same situation was shown in other cultivars such as benzeneacetaldehyde and (−)-myrtenol for Ayamurasaki; (E,E)-2,4-decadienal, hexanal, and (E)-2-hexenal for Jieshu95-16; and (E,Z)-2,4-decadienal, (E)-2-octenal, trans-β-ionone, nonanal, nerol oxide, geranylacetone, and 4-(1-methylethyl)-1,5-cyclohexadiene-1-methanol for Shuangzai. Furthermore, the GC-MS technology could easily distinguish the four cultivars and identify different compounds, which could further confirm the practicability of the optimized method.

## 3. Materials and Methods

### 3.1. Sample Preparation

Four sweet potato cultivars—Anna, Jieshu95-16, Ayamurasaki, and Shuangzai—were used as experimental plant materials in this study. All cultivars were planted on the 11 July 2020 using the standard production practices at the Baiyun Experimental Station (23°23′ N, 113°26′ E; 20 m above sea level) of the Guangdong Academy of Agricultural Sciences, Guangzhou, China. On 23 November 2020, triplicate samples were harvested. For each cultivar, 10 medium-sized tuberous roots were selected, washed with abundant tap water, rinsed with distilled water, dried with paper towel, cut into strips with a high-grade stainless knife, mixed well, weighed to 200 g, and then frozen immediately in liquid nitrogen. All of the samples were separately ground in a liquid nitrogen grinder (A10 basic, IKA, Staufen, Germany) and stored at −80 °C prior to GC-MS analysis. One gram of sweet potato sample was stored in a 15 mL headspace vial with 1 mL saturated NaCl solution added. Then, 5 μL of 1-pentanol solution (0.4055 mg mL^−^^1^) was added and the mixture was gently homogenized. The vial was capped with a crimp type cap and a PTFE/silicone septum (CNW, Shanghai, China) to prevent the leakage of volatile compounds.

### 3.2. Chemical and Reagents

All solvents used were chromatographic grade. 1-Pentanol, employed as an internal standard, and a mixture of *n*-alkanes (C7-C30) used for the retention index (RI) analyses were purchased from Sigma-Aldrich (Steinheim, Germany). NaCl of analytical grade was purchased from Guangzhou Chemical Reagent Factory (Guangzhou, China), and water was purified using a Milli-Q system (Millipore, Billerica, Massachusetts).

### 3.3. Fiber Selection

In this study, the manual sampling SPME holder was used and four different fused silica fibers (Supelco-Aldrich, Bellefonte, PA, USA) were tested: polydimethylsiloxane (PDMS) with 100 μm thickness, carboxen/polydimethylsiloxane (CAR/PDMS) with 75 μm thickness, polydimethylsiloxane/divinylbenzene (PDMS/DVB) with 65 μm thickness, and divinylbenzene/carboxen/polydimethylsiloxane (DVB/CAR/PDMS) with 50/30 μm thickness. Prior to the sampling, all the above fibers were conditioned using their recommended temperature and duration: PDMS, 250 °C for 30 min; CAR/PDMS, 300 °C for 30 min; PDMS/DVB, 230 °C for 30 min; and DVB/CAR/PDMS, 270 °C for 60 min.

Different fibers were first manually inserted into the sample vial that had been incubated in a dry heat block for 30 min at 80 °C, and exposed to the headspace of aqueous saline for the extraction at the same temperature for 30 min. After extraction, the fibers were immediately injected into the GC injection port for desorption at 250 °C for 3 min in splitless mode. The samples of sweet potato cultivar Anna were used to test the specific extraction capacity of fibers based on the total chromatographic area and number of chromatographic peaks.

### 3.4. Selection of Incubation Conditions

The optimized fiber coating was thereafter used to investigate the optimal incubation condition, which makes sweet potato samples reach the cooked and equilibrium state in the incubation process. Carbohydrate changes during sweet potato cooking are mainly due to starch degradation and maltose synthesis, β-amylase activity, and starch gelatinization temperature were the main regulatory factors in the process. Since β-amylase remains high activity between 60 °C to 80 °C and the gelatinization temperature of starch in sweet potato generally ranges from 53 °C to 80 °C [55], thus, three incubation temperatures (60 °C, 70 °C, and 80 °C) and three times of incubation (5 min, 10 min, and 30 min) were tested.

### 3.5. Selection of Extraction Time

Based on the optimized temperature and incubation time, three different extraction times of 30 min, 40 min, and 50 min were tested in order to evaluate the time required for volatile compounds to reach equilibrium on the matrix, solvent, and fiber coating [33].

### 3.6. GC-MS Analysis

The extracted volatile components trapped by the SPME fiber were desorbed in the GC injection port for 3 min at 250 °C in the splitless mode. GC-MS analysis was performed using an Aglient 7890B gas chromatograph system with a 5977B mass spectrometer (Agilent Technologies, CA, USA). A HP-5ms column (30 m, 250 μm, 0.25 μm) (Agilent Technologies, CA, USA) and a constant helium flow of 1 mL min^−1^ were used for chromatographic separation. The oven temperature program was initially 35 °C for 2 min, 5 °C min^−1^ ramp to 190 °C held for 1 min, 20 °C min^−1^ ramp to 250 °C held for 2 min, solvent delay of 1 min. The detector was operated in electron impact (EI) ionization mode at 70 eV in a full scan mode with a mass/charge ratio (m z^−1^) range from 35 to 450.

Chromatograms and mass spectra were analyzed using the Enhanced ChemStation software (Agilent Technologies, CA, USA). Identification of tentative volatile compounds was achieved by matching the mass spectra with the data system library (NIST 2017) and linear retention index (RI) sourced from the NIST Standard Reference Database. Aroma compounds in sweet potato were semi-quantitated using 1-pentanol solution (5 μL, 0.4055 mg mL^−1^) as an internal standard. The concentrations of aroma compounds were calculated on the basis of the ratio between peak area and the concentration of 1-pentanol. All the analyses were repeated in duplicate or triplicate.

### 3.7. Sensory Analysis

Sensory analysis was performed by ten well-trained panelists (five males and five females, with ages ranging from 20 to 45) from the Crops Research Institute at Guangdong Academy of Agricultural Sciences, China. Panelists each received a representative sample of the sweet potatoes and were trained to increase their sensitivity and ability to discriminate between the sensory attributes of the different cultivars. The whole process of sensory evaluation was performed at room temperature. The intact sweet potatoes were steamed for 45 min with a lid on the saucepan to prevent excessive moisture loss and then cut into 1 cm pieces for evaluation. In the process of evaluation, members were not allowed to communicate with each other, the taste of the sample could not be swallowed but spit out, and rinsed with water. The evaluations of each panelist were recorded on the questionnaire. Eight sensory descriptors involving firmness, aroma, sweetness, starchiness, viscosity, coarse texture, fibrous texture, and overall taste were selected to assess the sweet potato samples. The aroma intensity was described from 0 (not aromatic) to 3 (intensely aromatic), and the overall taste was evaluated out of a maximum score of 100. The intensity of other descriptors was evaluated on a 5-point scale from 0 (not perceivable) to 5 (strongly perceivable). Informed consent was obtained from all individual participants included in the study.

### 3.8. Volatile Analysis of Four Sweet Potato Cultivars

Volatile compounds of four sweet potato cultivars were analyzed by the optimized HS-SPME method: the samples in the vial were incubated in the dry heat block at 80 °C for 30 min and then extracted by the DVB/CAR/PDMS fiber in the headspace for 30 min. The volatiles in the fiber were used for GC-MS analysis (described in Section 2.5).

### 3.9. Statistical Analysis

Each cultivar was tested in triplicate to ensure the reliability of the experimental results. GC–MS results data were processed using IBM SPSS Statistics 24 (IBM Corp., Armonk, NY, USA) and represented as the mean ± standard deviation (SD). The results were statistically analyzed by one-way analysis of variance (ANOVA) and LSD multiple range test. A difference of *p* ≤ 0.05 was considered as significant. The unsupervised principal component analysis (PCA) with 95% confidence interval in the PCA score plot was used as the threshold to identify potential outliers in the dataset. The PCA was carried out using the *prcomp* and *ggplot2* package of R. The partial least squares-discrimination analysis (PLS-DA) was performed with MetaboAnalyst 5.0 (MetaboAnalyst). During the PLS-DA analysis, the GC-MS volatile data were pre-treated with auto scaling (mean-centered and divided by the standard deviation of each variable) and then used as the input value.

## 4. Conclusions

A HS-SPME/GC-MS method for detecting the volatile compounds in sweet potato was developed in this study. The optimized fiber was first selected with a DVB/CAR/PDMS coating that could absorb the largest number of identifiable peaks and highest peak intensity. The optimum HS-SPME conditions for the extraction were determined by studying the effect of incubation time, incubation temperature, and extraction time. The equilibrium condition among the sample, headspace, and fiber coating was provided by incubating the samples at 80 °C for 30 min and headspace extraction for 30 min at the same temperature. The proposed method was applied for the qualitative and semi-quantitative characterization of the volatile compounds from four sweet potato cultivars: Anna, Jieshu95-16, Ayamursaki, and Shuangzai.

Overall, 68 compounds in 11 chemical classes were identified in these four sweet potato cultivars using the optimized method. Aldehydes, alcohols, terpenes, and ketones dominated over the other classes. Among them, 23 volatiles were identified as common compounds of the four sweet potato cultivars. Differences in the kinds and contents of volatile compounds were revealed on the basis of their volatile profiles. In particular, Anna and Ayamurasaki contained higher levels of compounds with sweet, fresh, and fruity flavors whereas Jieshu95-16 and Shuangzai were more abundant in compounds that contributed to green and fat odor notes. The proposed method is simple, fast, and precise, and could serve as a useful tool to assess the aromatic profile of sweet potato. Furthermore, understanding the volatile components of sweet potato will be a good start point to explore the aromatic active compounds of sweet potato, which will be beneficial to the flavor improvement in sweet potato breeding. Nevertheless, the role of volatile compounds detected by GC-MS in sweet potato was unclear in sensory evaluation, so further study on the aroma impact of each compound and precise quantification are necessary.

## Figures and Tables

**Table 1:** Identification of volatile compounds in the four sweet potato cultivars.

Class	RT ^1^	RI ^2^	Compound	CAS	MF ^3^	ID Method ^4^	Flavor	Odor Thresholds (μg/g) ^5^	Relative Content (μg/g) ^6^			
									Anna	Jieshu95-16	Ayamursaki	Shuangzai
Aldehydes	3.44	676	Pentanal	000110-62-3	C5H10O	MS,RI	almond, malt, pungent	0.2	-	0.12 ± 0.05	-	-
	4.62	748	(E)-2-Pentenal	001576-87-0	C5H8O	MS,RI	strawberry, fruit, tomato	0.1	0.09 ± 0	-	-	-
	5.71	796	Hexanal	000066-25-1	C6H12O	MS,RI	grass, tallow, fat	0.21	1.29 ± 0.34	1.84 ± 0.47	1.12 ± 0.14	1.78 ± 0.24
	7.19	854	(E)-2-Hexenal	006728-26-3	C6H10O	MS,RI	apple, green	0.04	0.16 ± 0.01 ^c^	1.28 ± 0.34 ^a^	0.84 ± 0.23 ^a,b^	0.67 ± 0.13 ^b^
	8.67	901	Heptanal	000111-71-7	C7H14O	MS,RI	fat, citrus, rancid	0.031	0.62 ± 0.11 ^a^	0.4 ± 0.07 ^b^	0.16 ± 0.01 ^c^	0.59 ± 0.06 ^a^
	10.38	959	(E)-2-Heptenal	018829-55-5	C7H12O	MS,RI	soap, fat, almond	0.75	0.17 ± 0.01	0.24 ± 0.03	0.12 ± 0.01	0.33 ± 0.05
	10.49	963	Benzaldehyde	000100-52-7	C7H6O	MS,RI	almond, burnt sugar	1	0.85 ± 0.17 ^b^	1.08 ± 0.19 ^a,b^	1.32 ± 0.13 ^a^	0.75 ± 0.13 ^b^
	12.09	1012	(E,E)-2,4-Heptadienal	004313-03-5	C7H10O	MS,RI	nut, fat	0.03	0.15 ± 0.01 ^b^	0.36 ± 0.08 ^a^	0.22 ± 0.02 ^b^	0.19 ± 0.03 ^b^
	13.16	1048	Benzeneacetaldehyde	000122-78-1	C8H8O	MS,RI	honey, sweet	0.04	1.87 ± 0.52 ^b^	1.85 ± 0.49 ^b^	3.95 ± 0.64 ^a^	0.64 ± 0.15 ^c^
	13.61	1062	(E)-2-Octenal	002548-87-0	C8H14O	MS,RI	green, nut, fat	0.125	1.23 ± 0.06 ^c^	1.59 ± 0.03 ^b^	0.56 ± 0.09 ^d^	1.93 ± 0.2 ^a^
	15.01	1106	Nonanal	000124-19-6	C9H18O	MS,RI	fat, citrus, green	0.0035	-	0.2 ± 0.01	0.12 ± 0.01	0.97 ± 0.65
	17.12	1178	2,4-dimethylBenzaldehyde	015764-16-6	C9H10O	MS,RI	cherry almond vanilla	-	0.1 ± 0.04	-	-	-
	18.23	1216	(E,E)-2,4-Nonadienal	005910-87-2	C9H14O	MS,RI	fat, wax, green	0.0006	0.13 ± 0.01 ^b^	0.12 ± 0.02 ^b^	0.09 ± 0.01 ^b^	0.25 ± 0.05 ^a^
	18.47	1225	β-cyclocitral	000432-25-7	C10H16O	MS,RI	mint	-	0.08 ± 0.01	-	-	0.28 ± 0.02
	19.01	1244	Cuminaldehyde	000122-03-2	C10H12O	MS,RI	acid, sharp	0.4	0.24 ± 0.02 ^b^	0.16 ± 0.02 ^c^	0.35 ± 0.01 ^a^	0.23 ± 0.01 ^b^
	19.84	1274	Citral	005392-40-5	C10H16O	MS,RI	lemon	0.005	0.28 ± 0.02 ^a^	0.27 ± 0.05 ^a^	0.17 ± 0.04 ^b^	0.33 ± 0.04 ^a^
	19.97	1278	(−)-Perillaldehyde	002111-75-3	C10H14O	MS,RI	spicy, sweet, lime	0.0253	-	-	0.08 ± 0	-
	20.48	1295	(E,Z)-2,4-Decadienal	025152-83-4	C10H16O	MS,RI	fried fatty geranium	0.004	-	0.9 ± 0.21	0.76 ± 0.32	1.1 ± 0.28
	21.11	1320	(E,E)-2,4-Decadienal	025152-84-5	C10H16O	MS,RI	fried, wax, fat	0.0005	1.63 ± 0.29 ^a,b^	2.77 ± 0.91 ^a^	1.27 ± 0.26 ^b^	2.36 ± 0.68 ^a,b^
	23.26	1400	Vanillin	000121-33-5	C8H8O3	MS,RI	vanilla	0.03	0.1 ± 0.02 ^b^	0.14 ± 0.02 ^b^	0.24 ± 0.06 ^a^	0.13 ± 0.02 ^b^
	30.66	1715	Pentadecanal	002765-11-9	C15H30O	MS,RI	fresh waxy	-	-	0.17 ± 0.02	-	-
Alcohols	7.7	871	1-Hexanol	000111-27-3	C6H14O	MS,RI	flower, green	0.2	0.15 ± 0.03 ^b^	0.34 ± 0.12 ^a^	0.41 ± 0.04 ^a^	0.3 ± 0.02^a^
	11.13	982	1-Octen-3-ol	003391-86-4	C8H16O	MS,RI	mushroom	0.002	0.32 ± 0.04	-	-	-
	12.46	1025	3-Ethyl-4-methylpentan-1-ol	038514-13-5	C8H18O	MS,RI	-	-	0.08 ± 0.01	-	0.2 ± 0.01	-
	12.86	1038	Benzyl alcohol	000100-51-6	C7H8O	MS,RI	sweet, flower	5.5	0.27 ± 0.02	-	0.09 ± 0	0.18 ± 0
	15.28	1115	Phenylethyl Alcohol	000060-12-8	C8H10O	MS,RI	honey, rose, lilac	0.045	0.17 ± 0	0.09 ± 0.01	0.13 ± 0.02	-
	17.2	1181	Terpinen-4-ol	000562-74-3	C10H18O	MS,RI	turpentine, nutmeg, must	-	0.36 ± 0.06 ^a,b^	0.34 ± 0.15 ^a,b^	0.18 ± 0.08 ^b^	0.55 ± 0.06 ^a^
	17.59	1193	α-Terpineol	000098-55-5	C10H18O	MS,RI	lilac floral	0.3	-	-	0.24 ± 0	0.13 ± 0.02
	17.77	1199	(−)-Myrtenol	019894-97-4	C10H16O	MS,RI	pine balsam mint	-	0.58 ± 0.02 ^b^	0.19 ± 0.03 ^c^	0.82 ± 0.05 ^a^	0.17 ± 0.03^c^
	20.44	1294	*p*-Cymen-7-ol	000536-60-7	C10H14O	MS,RI	caraway-like herb	-	2.18 ± 0.14	-	0.66 ± 0.19	-
	20.64	1301	Perillyl alcohol	000536-59-4	C10H16O	MS,RI	orange peel floral	0.7	0.21 ± 0.01	-	0.11 ± 0.01	-
Ketones	3.27	662	1-Penten-3-one	001629-58-9	C5H8O	MS,RI	fish, pungent	0.001	-	0.11 ± 0.04	-	-
	8.35	891	2-Heptanone	000110-43-0	C7H14O	MS,RI	soap	0.68	0.11 ± 0.04	0.1 ± 0.03	-	0.1 ± 0.01
	11.1	981	1-Hepten-3-one	002918-13-0	C7H12O	MS,RI	metal	0.00004	-	0.28 ± 0.04	0.43 ± 0.06	-
	11.38	989	6-methyl-5-Hepten-2-one	000110-93-0	C8H14O	MS,RI	pepper, mushroom	0.1	-	-	-	0.1 ± 0.03
	13.97	1074	3,5-Octadien-2-one	038284-27-4	C8H12O	MS,RI	fruity fatty mushroom	0.3	-	0.11 ± 0.01	-	-
	16.07	1143	3-Nonen-2-one	014309-57-0	C9H16O	MS,RI	oily spicy waxy	-	-	0.15 ± 0.06	-	0.12 ± 0.02
	16.5	1158	(+)-Dihydrocarvone	005948-04-9	C10H16O	MS,RI	warm herbal	-	-	-	0.34 ± 0.08	-
	16.77	1167	Pinocarvone	030460-92-5	C10H14O	MS,RI	minty	-	-	0.06 ± 0	-	-
	24.62	1456	Geranylacetone	003796-70-1	C13H22O	MS,RI	floral fruity banana	0.01	-	-	-	0.31 ± 0.01
	25.48	1490	β-Ionone epoxide	023267-57-4	C13H20O2	MS,RI	fruit, sweet, wood	-	-	-	0.2 ± 0.06	-
	25.49	1490	Trans-β-Ionone	000079-77-6	C13H20O	MS,RI	violet, flower, raspberry	0.0005	0.51 ± 0.14	-	-	1.28 ± 0.13
Terpenes	12.53	1027	*p*-Cymene	000099-87-6	C10H14	MS,RI	Mild, pleasant; aromatic	0.0133	0.09 ± 0.02	-	-	0.17 ± 0.03
	12.75	1035	3-ethyl-2-methyl-1,3-Hexadiene	061142-36-7	C9H16	MS,RI	-	-	1.33 ± 0.17 ^a^	1.56 ± 0.17 ^a^	0.29 ± 0.04 ^b^	1.26 ± 0.16 ^a^
	14.88	1101	Linalool	000078-70-6	C10H18O	MS,RI	flower, lavender	0.0015	-	0.09 ± 0.02	0.08 ± 0.01	0.16 ± 0.02
	18.09	1210	Camphene	000076-22-2	C10H16O	MS,RI	Camphoraceous	0.88	2.34 ± 0.11 ^a^	0.43 ± 0.04 ^c^	1.69 ± 0.04 ^b^	0.62 ± 0.27 ^c^
	18.67	1232	Nerol	000106-25-2	C10H18O	MS,RI	sweet	0.08	0.71 ± 0.04 ^a^	0.16 ± 0.03 ^d^	0.41 ± 0.04 ^c^	0.58 ± 0.08 ^b^
	19.38	1258	Geraniol	000106-24-1	C10H18O	MS,RI	rose, geranium	0.0075	0.3 ± 0.01	0.26 ± 0.01	0.5 ± 0.03	-
	22.47	1372	(+)-Cyclosativene	022469-52-9	C15H24	MS,RI	-	-	0.29 ± 0.01	0.1 ± 0	-	-
	23.39	1406	Cyperene	002387-78-2	C15H24	MS,RI	-	-	0.11 ± 0	-	0.26 ± 0.05	0.08 ± 0
	24.33	1444	α-Guaiene	003691-12-1	C15H24	MS,RI	wood, balsamic	-	0.08 ± 0	-	-	-
	24.74	1461	Humulene	006753-98-6	C15H24	MS,RI	woody	0.16	0.12 ± 0	-	-	-
	25.55	1492	β-Selinene	017066-67-0	C15H24	MS,RI	herbal	-	-	0.14 ± 0.02	-	-
Acids	17.1	1177	Octanoic acid	000124-07-2	C8H16O2	MS,RI	rancid soapy fatty	0.8	-	0.38 ± 0.31	-	-
	27.25	1565	Dodecanoic acid	000143-07-7	C12H24O2	MS,RI	metal	-	0.14 ± 0.04	0.1 ± 0.02	-	0.11 ± 0.03
	34.72	1977	*n*-Hexadecanoic acid	000057-10-3	C16H32O2	MS,RI	odorless	-	3.88 ± 1.24 ^a^	2.92 ± 0.96 ^a,b^	1.75 ± 0.36 ^b^	3.03 ± 0.24 ^a,b^
Esters	26.58	1536	Dihydroactinidiolide	015356-74-8	C11H16O2	MS,RI	ripe apricot	0.5	0.38 ± 0.12	-	0.35 ± 0.13	0.21 ± 0.04
	34.26	1929	Hexadecanoic acid, methyl ester	000112-39-0	C17H34O2	MS,RI	oily waxy orris	-	0.17 ± 0.02	0.23 ± 0.06	0.11 ± 0.01	-
Alkanes	25.74	1500	Pentadecane	000629-62-9	C15H32	MS,RI	sweet creamy vanilla	-	-	-	0.09 ± 0	-
	35.71	2099	Heneicosane	000629-94-7	C21H44	MS,RI	waxy	-	0.06 ± 0.01	-	0.12 ± 0.05	-
Ethers	16.28	1150	1,2-dimethoxyBenzene	000091-16-7	C8H10O2	MS,RI	waxy	-	-	-	0.07 ± 0.01	-
Furan	11.52	993	2-pentylFuran	003777-69-3	C9H14O	MS,RI	green bean, butter	0.0048	0.74 ± 0.24 ^a^	0.64 ± 0.21 ^a,b^	0.33 ± 0.05 ^b^	0.88 ± 0.13 ^a^
Phenols	14.55	1091	Guaiacol	000090-05-1	C7H8O2	MS,RI	smoke, sweet, medicine	0.00017	0.43 ± 0.12 ^b^	0.1 ± 0.03 ^c^	0.97 ± 0.15 ^a^	0.36 ± 0.09 ^b^
	22.19	1361	Eugenol	000097-53-0	C10H12O2	MS,RI	clove, honey	0.001	0.07 ± 0	-	-	-
Others	4.57	745	1-chloroPentane	000543-59-9	C5H11Cl	MS,RI	sweet	640	-	-	-	0.1 ± 0.01
	16.52	1158	Nerol oxide	001786-08-9	C10H16O	MS,RI	oil, flower	0.08	-	-	-	0.37 ± 0.03
	17.67	1195	4-(1-methylethyl)-1,5-Cyclohexadiene-1-methanol	019876-45-0	C10H16O	MS,RI	-	-	-	0.09 ± 0.01	-	0.3 ± 0.05
	24.8	1463	6-pentyl-2H-Pyran-2-one	027593-23-3	C10H14O2	MS,RI	sweet creamy coconut	-	-	0.15 ± 0.04	-	-

^1^ RT: retention time; ^2^ RI: line retention indices; ^3^ MF: molecular formula; ^4^ MS: identification by comparison with mass spectra; RI: identified by retention indices; ^5^ The odor thresholds were taken from the literature and were values in water [39]. ^6^ The relative content was represented as relative concentration, calculated by the peak area ratio between each compound and the internal standard and the concentration of the internal standard. Different uppercase letters (a, b, c, d) in the same line indicate significant differences among cultivars (*p* < 0.05).

**Table 2:** Classes of volatiles characterizing four different sweet potato cultivars.

Class	Anna	Jieshu95-16	Ayamurasaki	Shuangzai
Aldehydes	8.95 ± 0.56 ^c^	13.27 ± 0.93 ^a^	11.09 ± 0.417 ^b^	12.23 ± 0.97 ^a,b^
Alcohols	4.31 ± 0.23 ^a^	0.95 ± 0.24^c^	2.19 ± 0.32 ^b^	1.32 ± 0.07 ^c^
Ketones	0.62 ± 0.19 ^b^	0.70 ± 0.16 ^b^	0.9 ± 0.0.21 ^b^	1.9 ± 0.12 ^a^
Terpenes	5.23 ± 0.16 ^a^	2.62 ± 0.19 ^b^	3.24 ± 0.08 ^b^	2.87 ± 0.38 ^b^
Acids	4.02 ± 1.23 ^a^	3.28 ± 0.66 ^a,b^	1.75 ± 0.36 ^b^	3.14 ± 0.21 ^a,b^
Esters	0.55 ± 0.1 ^a^	0.23 ± 0.05 ^b^	0.46 ± 0.13 ^a^	0.21 ± 0.03 ^b^
Furan	0.74 ± 0.0.23 ^a^	0.64 ± 0.21 ^a,b^	0.33 ± 0.0.05 ^b^	0.88 ± 0.13 ^a^
Phenols	0.47 ± 0.08 ^b^	0.1 ± 0.03 ^c^	0.97 ± 0.14 ^a^	0.36 ± 0.09 ^b^
Alkanes	0.06 ± 0	-	0.21 ± 0.05	-
Ethers	-	-	0.07 ± 0	-
Others	0.08 ± 0.01 ^c^	0.21 ± 0.08 ^b^	0.11 ± 0.01 ^c^	0.77 ± 0.02 ^a^
Total content	24.98 ± 0.21 ^a^	21.67 ± 0.42 ^c^	21.33 ± 0.55 ^c^	23.4 ± 0.4 ^b^

The value of each volatile class was represented by the mean of relative concentration (μg/g) ± standard deviation, calculated by the peak area ratio between each compound and the internal standard, and the concentration of the internal standard. Different uppercase letters in the same line indicate significant differences among cultivars (*p* < 0.05).

## Data Availability

The data presented in this study are available in article and Appendix A.

## Appendix A

**Table A1:** Comprehensive sensory evaluation of sweet potato.

Variety	Firmness	Aroma	Sweetness	Starchiness	Viscosity	Coarse Texture	Fibrous Texture	Overall Taste
Ayamurasaki	2.43 ^a^	2.14 ^a^	2.86 ^a^	2.43 ^a^	2 ^a^	2.43	0.57 ^a,b^	88.71 ^a^
Anna	1.23 ^b^	2.07 ^a^	3.43 ^a^	2.43 ^a^	2.14 ^a^	1.64	0.14 ^b^	89.79 ^a^
Jieshu95-16	2.05 ^a^	1.73 ^b^	1.73 ^b^	2.64 ^a^	1.55 ^a^	1.36	1.18 ^a^	77 ^b^
Shuangzai	1.07 ^b^	0.8 ^c^	0.8 ^c^	0.4 ^b^	0.6 ^b^	1.2	0.47 ^a,b^	63.53 ^c^

Means with different lowercase letters were significantly different at *p* < 0.05.
